# A Meta-Analysis of Comparing Intermittent Epidural Boluses and Continuous Epidural Infusion for Labor Analgesia

**DOI:** 10.3390/ijerph17197082

**Published:** 2020-09-27

**Authors:** I-Shiang Tzeng, Ming-Chang Kao, Po-Ting Pan, Chu-Ting Chen, Han-Yu Lin, Po-Chun Hsieh, Chan-Yen Kuo, Tsung-Han Hsieh, Woon-Man Kung, Chu-Hsuan Cheng, Kuo-Hu Chen

**Affiliations:** 1 Department of Research, Taipei Tzu Chi Hospital, Buddhist Tzu Chi Medical Foundation, New Taipei City 23142, Taiwan; cykuo863135@gmail.com (C.-Y.K.); b87404037@gmail.com (T.-H.H.); 2 Department of Statistic, National Taipei University, Taipei 10478, Taiwan; 3 Department of Applied Mathematics; Department of Exercise and Health Promotion, Chinese Culture University, Taipei 11114, Taiwan; 4 Department of Anesthesiology, Taipei Tzu Chi Hospital, Buddhist Tzu Chi Medical Foundation, New Taipei City 23142, Taiwan; dr_mck@yahoo.com.tw (M.-C.K.); snakepan00@gmail.com (P.-T.P.); echoct@gmail.com (C.-T.C.); bbkeric@gmail.com (H.-Y.L.); 5 School of Medicine, Tzu Chi University, Hualien 97004, Taiwan; 6 Department of Chinese Medicine, Taipei Tzu Chi Hospital, Buddhist Tzu Chi Medical Foundation, New Taipei City 23142, Taiwan; charredalex@gmail.com; 7 School of Post-Baccalaureate Chinese Medicine, Tzu Chi University, Hualien 97004, Taiwan; 8 Division of Neurosurgery, Department of Surgery, Taipei Tzu Chi Hospital, Buddhist Tzu Chi Medical Foundation, New Taipei City 23142, Taiwan; nskungwm@yahoo.com.tw; 9 Department of Nursing, Taipei Tzu Chi Hospital, Buddhist Tzu Chi Medical Foundation, New Taipei City 23142, Taiwan; forcechoas@gmail.com; 10 Department of Obstetrics and Gynecology, Taipei Tzu Chi Hospital, Buddhist Tzu Chi Medical Foundation, New Taipei City 23142, Taiwan

**Keywords:** continuous epidural infusion (CEI), intermittent epidural bolus (IEB), labor analgesia, meta-analysis

## Abstract

With the development of medical equipment and techniques in labor anesthesia, it is a major issue to investigate the risks and treatment effects among techniques such as continuous epidural infusion (CEI) and intermittent epidural bolus (IEB). However, there is a controversial result regarding two techniques. This study was conducted through meta-analysis of randomized controlled trials (RCTs) for labor analgesia between the CEI and IEB techniques. The pooled results were presented as weighted mean differences (WMDs) together with 95% confidence intervals (CIs) and odds ratios (ORs) together with 95% CIs, respectively. Eleven RCTs were included in this meta-analysis. Four hundred sixty-five parturients accepted CEI, whereas 473 parturients accepted IEB labor analgesia. Elven identified low- risk bias studies were recruited for meta-analysis. The results presented no statistical difference in cesarean delivery rate between IEB and CEI (OR, 0.96; 95% CI, 0.67–1.37) and duration of second stage of labor (WMD, −3.82 min; 95% CI, −8.28 to 0.64). IEB had statistically significant lessened risk of instrumental delivery (OR, 0.59; 95% CI, 0.39–0.90) and for the use in local anesthetic (WMD, −1.71 mg bupivacaine equivalents per hour; 95% CI, −1.88 and −1.55). Accepted IEB had a higher score of maternal satisfaction (WMD, −6.95 mm; 95% CI, −7.77 to −6.13). Based on evidence, IEB showed a greater benefit for slightly reducing the use in local anesthetic, reduced risk of instrumental delivery, and improved maternal satisfaction for the requirement of labor epidural analgesia for healthy women. In the future, more studies need to be conducted to practice the IEB regimen and explore its influence on labor analgesia.

## 1. Introduction

The chronic pain associated with pregnancy has been understood [1]. It seems that during pregnancy, it is safe to use medications that are used in therapeutic doses for chronic pain. Chronic pain may accompany women until childbirth. The importance of managing chronic pain during pregnancy by obstetrical providers is well known. In the meantime, obstetrical providers are also faced with labor pain for parturients. In the early period, regarding complement of the second stage of labor, the process of epidural labor analgesia principally consists of a single injection of local anesthetic via an epidural needle. Compared with the total labor time, lessened pain relief duration ordinarily limits the type of analgesia. Childbirth is potentially treated as the suffering experience of a parturient [2]. Physicians frequently use high doses of local anesthetics to maintain analgesia; however, this poses increased risk of maternal hypotension, local anesthetic toxicity, and motor block of the lower body. This also causes a deficit in the pushing efficiency of the parturient. Considering the limited impact on delivery mode and on maternal and infant outcomes and the quality of labor neuralgia analgesia, traditional gastrointestinal opioids, nitrous oxide, and non-pharmacological measures are no longer adequate for use [3].

Epidural catheters became popular during the mid-1970s. In the 1980s, low densities of local anesthetic with continuous epidural infusion (CEI) became possible to give to parturients, with or without an opioid (i.e., fentanyl or sufentanil). Uneven analgesia and possible local toxicity could be avoided through the clinician’s management of intermittent bolus techniques. Patient-controlled epidural analgesia (PCEA) for labor pain was described by Gambling et al. in 1988 [4]. The progression of labor allowed the patient to match the dose of analgesia to the pain. It also allowed variability in patient dosage requirements. Most North American and European institutions undertake standard labor epidural analgesic regimens including local anesthetics combined with an opioid given via CEI with or without PCEA boluses. Although compared with non-neuraxial analgesia, CEI with or without PCEA has better analgesic effects, as high doses of local anesthetic doses lead to profound motor blockade [5]. There is a decreased ability to move, decreased pelvic floor muscle tension, and inability to hold down during the second stage of labor. Hence, it leads to an increase in the rate of dystocia and instrumental deliveries [6].

In addition, the data of the comparisons between a new technique used to keep labor analgesia, programmed intermittent epidural boluses (PIEB) CEI for analgesia in labor are conflicting. Due to the continuing evolution in labor analgesia, small regularly spaced intermittent boluses may cause the local anesthetic to spread more widely in the epidural space [7], instead of delivering the local anesthetic continuously. Therefore, administering the same local anesthetic dosage by intermittent epidural bolus (IEB) or PIEB may improve the analgesic effect. With the development of new medical equipment, research on IEB is also growing. To our knowledge, obstetric anesthesiologists permit an enhanced pump in order to allow a shift from CEI to IEB. Previous systematic reviews have discussed alternate forms of neuraxial analgesia [8,9,10] and labor epidural analgesia versus no analgesia. A previous systematic review compared traditional PCEA and CEI [11], which used the IEB dose at the time but is now out of date. This study conducted to compare the effects of CEI and IEB techniques in healthy women accepting labor epidural analgesia with or without PCEA by reviewing randomized controlled trials (RCTs). Moreover, we evaluated patient satisfaction, manual anesthesia interventions, progression of labor, and delivery mode among CEI and IEB.

## 2. Materials and Methods

### 2.1. Identification of Relevant and Eligible Studies

**Figure 1:**
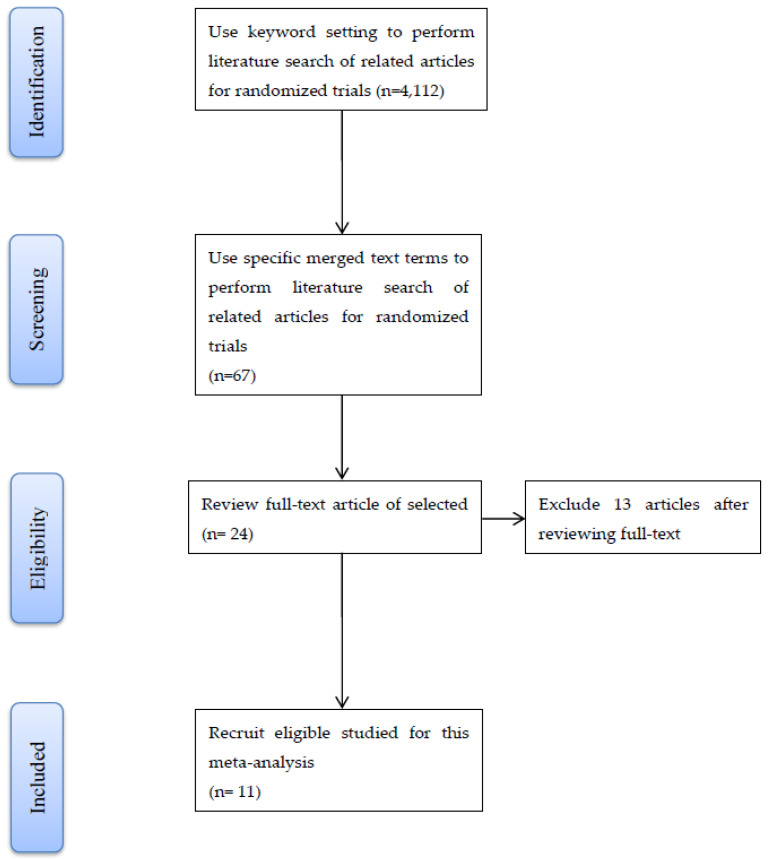
Flow chart of the selected eligible studies.

This section is a description obeying the preferred reporting items for systematic review and meta-analysis (PRISMA) [12]. Relevant literature was searched independently by two investigators using the PubMed, EMBASE, EBSCO, Springer Link, and Web of Science databases until July 2020. The literature search adopted the following keywords including: analgesia, epidural, anesthesia, patient-controlled, pregnancy, and obstetrical. Merged specific text terms were used to search for “intermittent” and “automated” and related synonyms. Human case-control studies were prepared as references. The types of studies were chosen by reviewing RCTs to compare the impact on CEI and IEB techniques for labor epidural analgesia with or without PCEA. Figure 1 presents the details of the study selection process as the PRISMA flowchart. The initial electronic database search yielded 4,112 records and 4,045 records were excluded regarding duplicated topics. A total of 43 irrelevant studies were removed after reviewing the title and abstracts. The 13 articles were excluded regarding lack of RCT design after reviewing the full-text.

### 2.2. Procedure of Extraction and Assessment of Eligible Studies

Two investigators (I.-S.T. and M.C.K.) independently evaluated qualified articles and collected the required data. A third reviewer (P.T.P.) helped to resolve discrepancies in the viewpoints of the two investigators. Hozo et al. [13] summarized converting formulas for three scenarios defined as C1, C2, and C3, which involved median, ranges, and interquartile range. We used a valid and appropriate converting formula to convert the mean and standard deviation, while extracted data were presented in terms of medians, ranges, and interquartile ranges. In addition, the standard deviation could also be converted through confidence intervals (CIs). Based on the criteria from Furlan et al. [14], all included studies were individually identified by two reviewers (C.T.C. and H.Y.L.) whether it had low risk bias quality. Twelve criteria were indicated as positive, negative, and inconclusive and they defined studies as low risk of bias if studies reached equal or more than six positive confirmations. The suggestion of the third reviewer (P.C.H.) was considered if the event of identification was inconsistent between I.-S.T. and M.C.K.

Extracted data relating to the pooled outcome were imported into STATA software (Version MP/14.0, Stata Corporation, College Station, TX, USA). A pooled model with random effects was conducted to explore the impact on labor analgesia among IEB and CEI. Continuous data were presented as weighted mean difference (WMD) together with 95% CI. Categorical data were presented as odds ratios (OR) together with 95% CI. Since heterogeneity does not equal chance [15], we presented the I^2^ statistic for the description of heterogeneity of each pooled model. A statistically significant difference was identified if a *p* value was <0.05 and I^2^ > 50% was used as a criterion to identify significant heterogeneity. Begg’s test [16] was adopted to identify publication bias.

## 3. Results

Figure 1 summarizes the study selection procedure. Eleven articles [17,18,19,20,21,22,23,24,25,26,27] were recruited in this study after screening 24 articles. All 13 studies were excluded because of a non-randomized comparison of CEI and IEB [28,29,30,31,32,33,34,35,36,37,38,39,40]. While 473 parturients received IEB labor analgesia, 465 parturients received CEI. Summary of the relevant characteristics of eligible studies are indicated in Supplementary Table S1. The results of the assessment of risk of bias are summarized in Supplementary Table S2. Finally, 11 studies were identified to be of low risk of bias. In the meantime, four studies [17,18,22,24] involved spontaneous onset of labor. Moreover, three studies presented a percentage of women who accepted preanalgesia oxytocin [18,22,24]. Four studies reported the proportion of women who had induced labor for childbirth [19,23,26,27]. Four studies reported the use of preanalgesia of oxytocin [20,21,26,27]. We summarized the general characters and meta-analysis results of nine pooled outcomes based on 11 eligible studies in Table 1.

### 3.1. Delivery Mode

**Figure 2:**
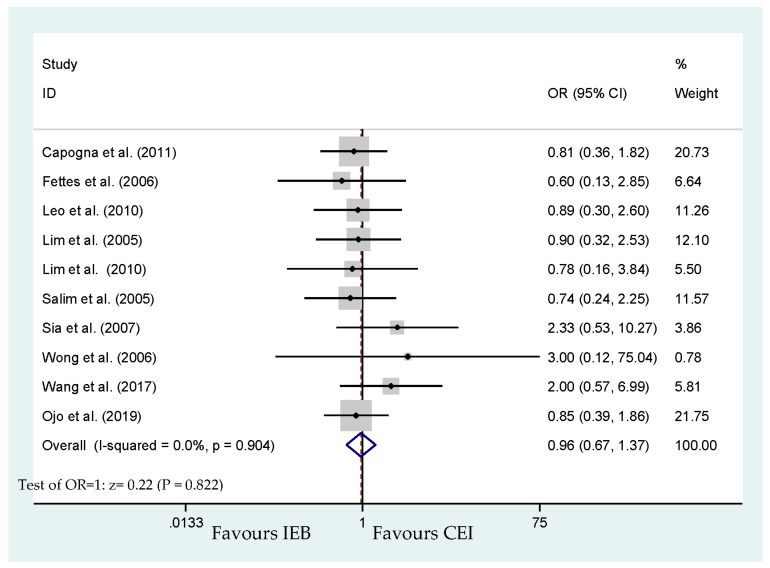
Overall effect for cesarean delivery mode.

**Figure 3:**
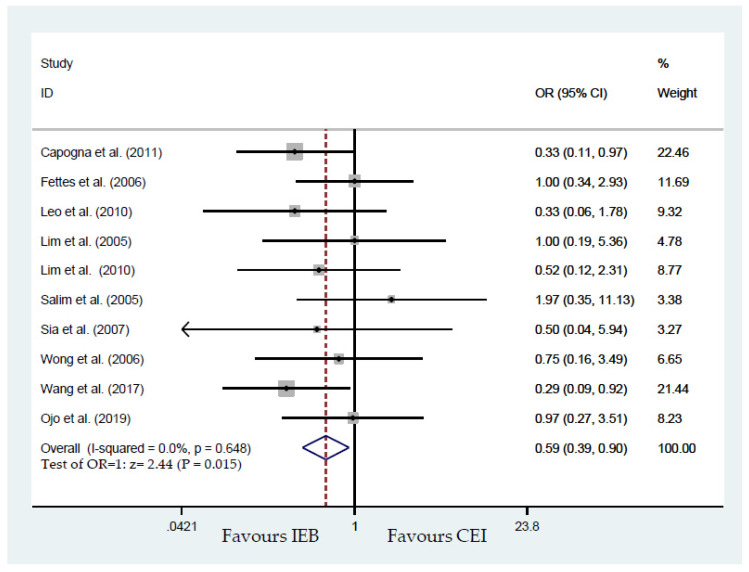
Overall effect for instrumental delivery mode.

Of the 11 included trials, the 10 studies that reported the mode of delivery included 896 parturients [17,19,20,21,22,23,24,25,26,27]. Pooled results showed insignificantly different cesarean delivery rate (OR, 0.96; 95% CI, 0.67–1.37; Figure 2). Begg’s test analysis showed no publication bias for cesarean delivery (*p* = 0.371). Similarly, in the studies analyzed by Capogna et al., a decrease in instrumental delivery rate was not reported [17]. Capogna et al. reported a significant lessening in instrumental delivery rates with IEB compared with CEI (7% vs. 20%; *p* = 0.03) [17]. IEB significantly reduced the risk of instrumental delivery based on the pooled model including 10 studies (OR, 0.59; 95% CI, 0.39–0.90; *p* = 0.015; Figure 3). No publication bias for instrumental delivery was detected by Begg’s test (*p* = 0.474).

### 3.2. Labor Duration

**Figure 4:**
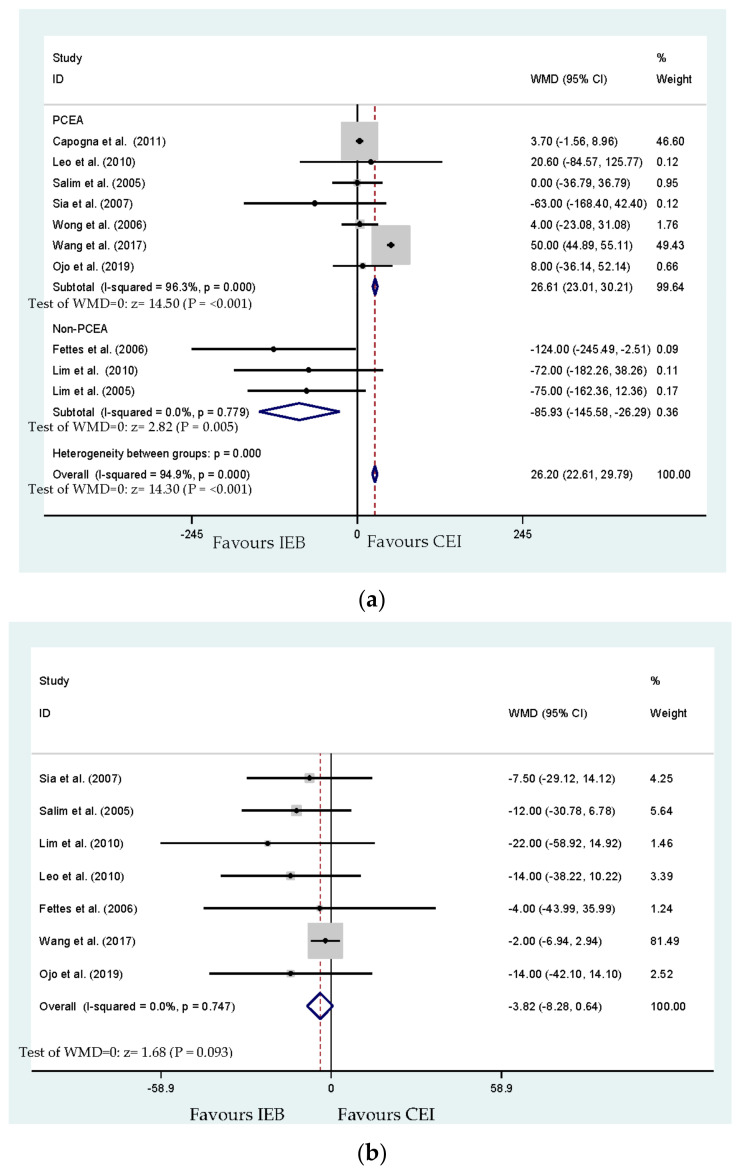
(**a**)**.** Effect of overall and subgroup analysis of the total duration (minutes) of labor analgesia. PCEA: Patient-controlled epidural analgesia. (**b**) Overall effect for the duration (minutes) of the second stage of labor analgesia.

The duration of the second stage of labor was reported in seven studies (n = 565) [19,20,22,23,24,26,27]; the total labor duration or labor analgesia duration was reported in 10 studies (n = 896) [17,19,20,21,22,23,24,25,26,27]. The results showed a statistically significant difference in the total duration of labor between IEB and CEI (*p* < 0.001; Figure 4a). Begg’s test analysis showed no publication bias for the duration of labor (*p* = 0.371). The results presented no statistically significant shortening of the duration of the second stage of labor in the IEB group (WMD, −3.82 min; 95% CI, −8.28 to 0.64; Figure 4b). No publication bias for the duration of the second stage of labor detected by Begg’s test (*p* = 0.764).

### 3.3. Intervention of Anesthesia

**Figure 5:**
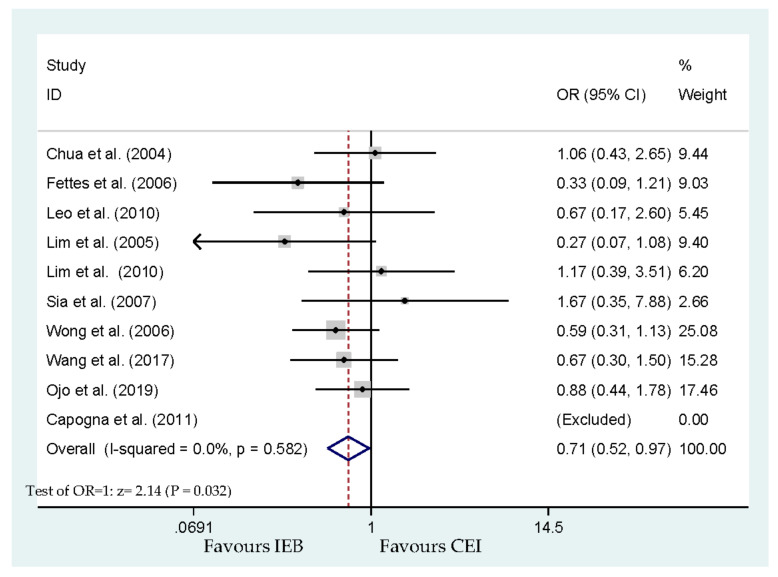
Overall effect for the requirement of anesthetic interventions.

**Figure 6:**
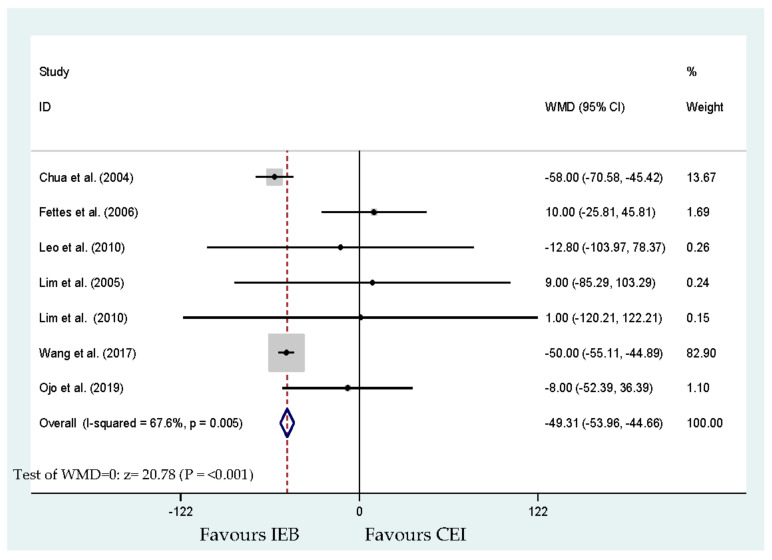
Overall effect for time (minutes) to first requirement of anesthetic intervention.

Ten studies reported that the anesthesia caregiver approved the requirement for manual boluses of local anesthetic [17,18,19,20,21,22,24,25,26,27]. Eight hundred and eleven parturients were involved in these 10 studies (Figure 5). The results showed a significant lessening in the requirement of extra anesthetic intervention (OR, 0.71; 95% CI, 0.52–0.97; *p* = 0.032; Figure 5). Begg’s test analysis showed no publication bias for manual interventions (*p* = 0.754). The was a significant difference between the time to the parturient’s first anesthetic intervention among the groups (WMD, −49.31 min; 95% CI, −53.96 to −44.66; *p* < 0.001; Figure 6). The time to the first intervention outcome also presented significant heterogeneity (I^2^ = 67.6%). The studies of Chua et al. [18] and Wang et al. [26] seemed to significantly increase the heterogeneity in this analysis. Two studies did not report the time to first anesthesia intervention [24,25]. Among CEI and IEB, there was no significant difference in the pain-free interval reported in a previous study [24]. The comparison of the time to first use of PCEA between the IEB and CEI groups showed a significant difference in time to intervention outcome [25]. No publication bias for time to intervention was detected by Begg’s test (*p* = 0.764).

### 3.4. Local Anesthetic Dosage

**Figure 7:**
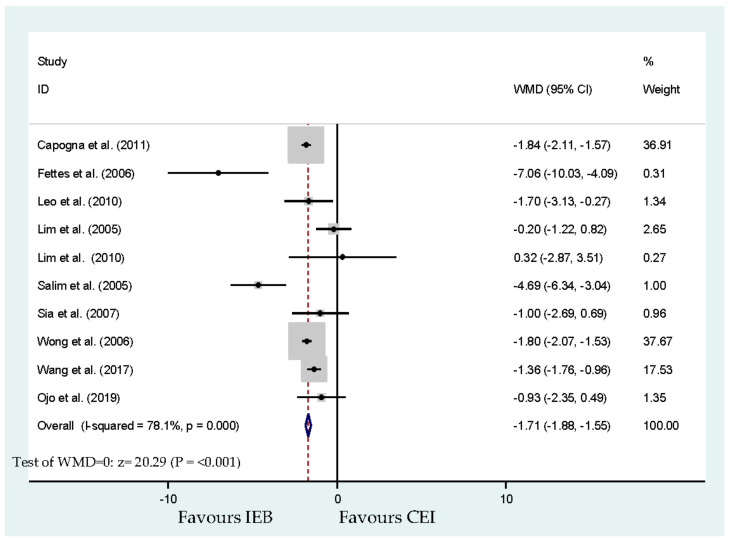
Overall effect for delivered dosage (milligrams per hour) of local anesthetic (bupivacaine equivalents).

Table 1 lists the specific treatments and local anesthetic concentrations. Among the 11 included studies, the total dose of local anesthetic was extracted from published studies [20,22,25,26]. The total dose was reported in six relevant studies [17,19,21,23,24,27]. A previous study [18] was excluded from this meta-analysis due to the conclusion that anesthetic intervention was first required. Eight hundred and ninety-seven parturients were involved in these 10 studies, showing a significant lessening in total local anesthetic delivered with IEB (WMD, −1.71 mg bupivacaine equivalents per hour; 95% CI, −1.88 and −1.55; I^2^ = 78.1%; *p* < 0.001; Figure 7). No publication bias for local anesthetic consumption was detected by Begg’s test (*p* = 0.858).

### 3.5. Maternal Satisfaction

**Figure 8:**
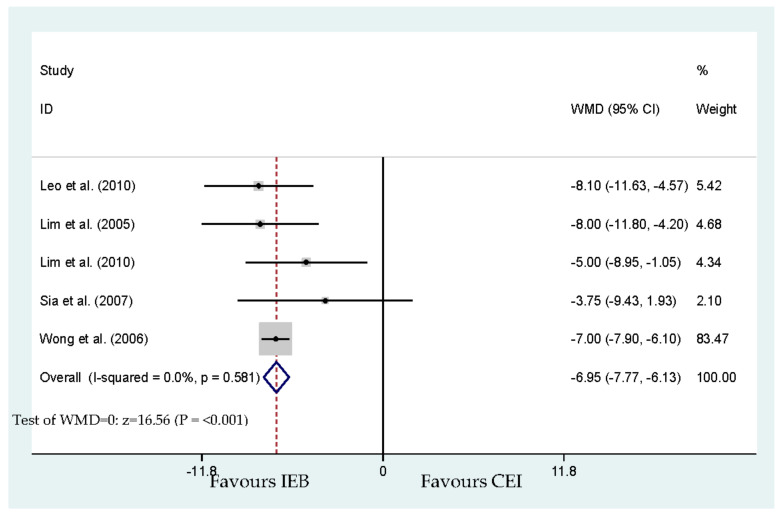
Overall effect for maternal satisfaction (visual analog scale from 0 to 100).

Five of the included studies [20,21,22,24,25] reported the overall maternal satisfaction with labor analgesia. A verbal rating scale (VRS) from 0 (dissatisfied) to 100 (extremely satisfied) was reported in four studies [20,21,22,24]. A 100-mm visual analog scale (VAS) score also reported maternal satisfaction in the study of Wong et al. [25]. For the IEB groups, pooled data retracted from the five studies presented higher maternal satisfaction (WMD, −6.95 mm; 95% CI, −7.77 and −6.13; Figure 8). No publication bias for maternal satisfaction was detected by Begg’s test (*p* = 0.221).

### 3.6. Subgroup Analysis Using PCEA

**Figure 9:**
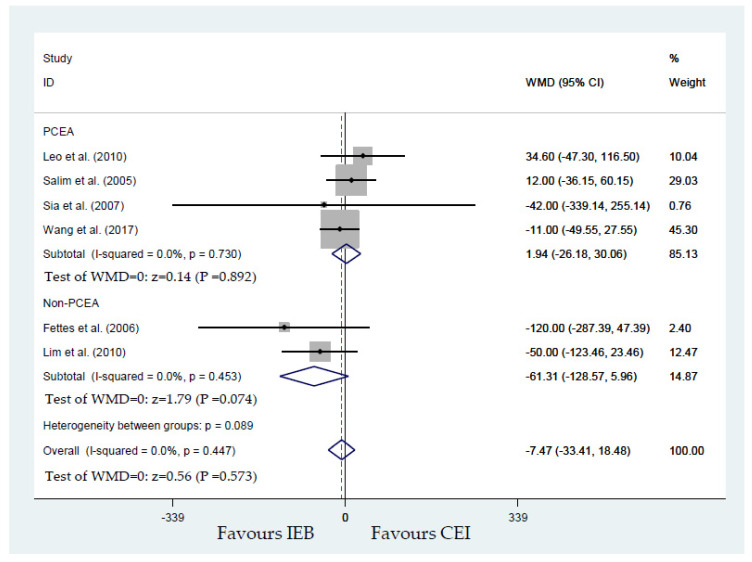
Overall effect for the overall and subgroup analysis of duration (minutes) of the first stage of labor analgesia.

According to the usage of PCEA or non-PCEA to maintain labor analgesia, subgroup analyses were performed for each primary outcome. The results of the pooled model of the initial use of PCEA to maintain labor analgesia showed a significant impact on the first stage labor duration and the total labor duration from four studies [20,23,24,26]. IEB provides a longer duration of the first stage while using PCEA to maintain labor [20,23,24,26] (WMD, 1.94 min; 95% CI, −26.18 to 30.06). In contrast, IEB may shorten the duration of the first stage of labor, while PCEA was not adopted to maintain labor [19,22] (WMD, −61.31 min; 95% CI, −128.57 to 5.96; Figure 9). No publication bias for the duration of the first stage of labor was detected by Begg’s test (*p* = 0.999).

## 4. Discussion

This study was based on the 11 recruited eligible low risk bias RCTs conducted on laboring women between the CEI and IEB techniques. The analyses involved 937 parturients from 11 RCTs well-conducted based on parturient characteristics. Concerning the requirement of labor epidural analgesia for healthy women, this meta-analysis study showed that IEB had a greater benefit to the parturient and fetus.

However, reduced local anesthetic consumption, improvement in maternal satisfaction, and decreased anesthetic interventions may be associated with IEB of local anesthetic compared with women accepting CEI. From a clinical viewpoint, the IEB treated was comparable with CEI for local anesthesia techniques. The total duration of labor between the two techniques were significantly different, but there was an insignificant lessening in the length of the second stage of labor. The CEI technique had a shorter total duration of labor as high as 26.2 min.

Although statistical significance was not reached, several results that could be treated involved clinical significance. The pooled model for cesarean delivery mode, the duration of the first stage of labor, and the duration of the second stage of labor involved wide CIs that contained clinically significant end points, so we could not draw conclusions on these results from the pooled data. For example, the lower end CI for the WMD for the duration of the first stage of labor was −34.41, which may have significance with IEB.

Instrumental deliveries may potentially cause labor analgesia. At the beginning of the active stage of labor, neuraxial analgesia needs to be withheld to prevent adverse labor outcomes [41]. It is worth noting that IEB delivery of epidural analgesia significantly affected labor outcomes and even the improvement in maternal satisfaction. For labor analgesia research, it is still critical to improve satisfaction through analgesia for labor.

An important result of the quality of women’s care is usually based on satisfaction with labor analgesia. We believe that this is different from describing a measure of adequacy of the overall pain relief. However, maternal decision-making sharing, consciousness of emotional control, maternal expectations, and labor pain could affect maternal satisfaction [42,43,44]. In addition, labor pain can reflect whether inadequately validated methods of maternal satisfaction included VAS and VRS measures. Sometimes, VAS and VRS measures cannot correspond to a certain level of the pain relief effect [45,46]. Heterogeneity may be caused by the different measuring satisfaction scales in the pooled results, which should be interpreted with caution.

The sample sizes of all of the studies were small, reflecting that they were powered to detect differences in outcomes. There were two trials powered to identify differences in the delivered dosage of local anesthetics [24,25]. Four hours after the intervention, Fettes et al. [19] provided a method to identify differences in VAS scores at any time. Four studies were aimed at detecting differences in the requirement of anesthesia interventions [18,20,21,22]. Wang et al. used VAS 0–10 cm to measure maternal satisfaction [26] and detected a significant difference in VAS score after the intervention. Due to the different scales of VAS [26], we did not undertake the pooled results of maternal satisfaction in this study.

The difference in treatment efficacy between IEB and CEI for labor analgesia has been addressed in previous systematic reviews and meta-analyses [47,48,49]. In 2013, a well-conducted meta-analysis [47] of nine studies that showed minor reduction in local anesthetic usage and improvement of maternal satisfaction were related to receiving IEB. Recently in 2019, a meta-analysis [48] included 11 studies that found that IEB with PCEA may critically reduce the incidence of instrumental delivery, severe pain, PCEA received rates, and local anesthetics utilization. Specifically, for the IEB with PCEA group, the results showed shorter labor duration and maternal satisfaction improvement compared to the CEI with PCEA group. The latest and final meta-analysis [49] recruited 22 RCTs with 2573 parturients. Among these meta-analyses, Liu et al. evidently conducted a meta-analysis with the largest sample size. However, a relevant and most recent study was excluded [27], and the results need to be updated. Hence, the current meta-analysis was constructed by recruiting RCTs to investigate the difference in efficacy between IEB and CEI techniques for labor analgesia.

In a nutshell, IEB is related to reduced local anesthetic consumption, shorter time to first required anesthetic intervention, and higher maternal satisfaction, and possibly a lessening in the risk of instrumental delivery. Xu et al. [48] found that PIEB plus PCEA may reduce the risk of instrumental delivery compared with CEI plus PCEA (OR, 0.51; 95% CI, 0.30–0.84, *p* = 0.009). Our result of IEB on the risk of instrumental delivery (OR, 0.59; 95% CI, 0.39–0.90, *p* = 0.015) was similar to the overall effect under PIEB plus PCEA (OR, 0.51; 95% CI, 0.30–0.84, *p* = 0.009). As above-mentioned, the pooled results, together with the wider CIs for some reported outcomes, may imply significant differences. For example, IEB may have a clinically significant effect on the first stage duration of labor analgesia. It is necessary to conduct more IEB research on labor analgesia before definitive conclusions are made.

This study has some limitations. First, there were significant inconsistent reported outcomes. At least one primary outcome of each study was included in this meta-analysis, but not all primary outcomes were reported in this meta-analysis. Second, as aforementioned, the overall effects should be carefully interpreted because of the lowest heterogeneity (I^2^ = 0%). Third, the mean and standard deviations were converted through converting formulas [13], while those RCTs presented median, range, and interquartile range. This means that there was a conversion bias involved in the pooled results in this meta-analysis. Fourth, trial sequential analysis [50] could be utilized to examine the reliability of the results of this meta-analysis with a small sample size. Sixth, weighting bias may occur due to some small trials given disproportionately large weights. Finally, publication bias may still exist due to the eligible studies published in English and Chinese.

## 5. Conclusions

In conclusion, current evidence shows that IEB has a greater benefit for slightly reduced local anesthetic usage, reduced risk of instrumental delivery, and improved maternal satisfaction on requirement of labor epidural analgesia for healthy women. The pooled results of many outcomes involved wide CIs, which means that it is impossible to draw a clear conclusion for those outcomes; however, IEB may potentially improve instrumental delivery rate and requirement of anesthesia interventions. In the future, more studies need to be conducted for the clinical practice of the IEB regimen and to explore its influence on labor analgesia.

## Figures and Tables

**Table 1:** Summary of general characteristics and results of pooled outcomes based on eligible studies.

Outcomes\Characteristics	Included Studies	No. of Participants	Overall WMD or OR and 95% CI	*p*-Value	I^2^ (%)	Forest Plot	Subgroup Analysis Effect (*p*-Value)
Cesarean delivery mode	Capogna et al. [17], Fettes et al. [19], Leo et al. [20], Lim et al. [21], Lim et al. [22], Salim et al. [23], Sia et al. [24], Wong et al. [25], Wang et al. [26], and Ojo et al. [27]	896	0.96 (0.67–1.37)	0.822	0.0	Figure 2	No
Instrumental delivery mode	Capogna et al. [17], Fettes et al. [19], Leo et al. [20], Lim et al. [21], Lim et al. [22], Salim et al. [23], Sia et al. [24], Wong et al. [25], Wang et al. [26], and Ojo et al. [27]	896	0.59 (0.39–0.90)	0.015 *	0.0	Figure 3	No
Total duration (minutes) of labor analgesia	Capogna et al. [17], Fettes et al. [19], Leo et al. [20], Lim et al. [21], Lim et al. [22], Salim et al. [23], Sia et al. [24], Wong et al. [25], Wang et al. [26], and Ojo et al. [27]	896	26.20 (22.61–29.79)	<0.001 *	94.9	Figure 4a	26.61 (<0.001 *) for PCEA ([17], [20], [23], [24], [25], [26], and [27]) −85.93 (0.005 *) for non-PCEA ([19], [21], and [22])
Duration (minutes) of second stage of labor analgesia	Fettes et al. [19], Leo et al. [20], Lim et al. [22], Salim et al. [23], Sia et al. [24], Wang et al. [26] and Ojo et al. [27]	565	−3.82 (−8.28 to 0.64)	0.093	0.0	Figure 4b	No
Requirement of anesthetic interventions	Capogna et al. [17], Chua et al. [18], Fettes et al. [19], Leo et al. [20], Lim et al. [21], Lim et al. [22], Sia et al. [24], Wong et al. [25], Wang et al. [26] and Ojo et al. [27]	811	0.71 (0.52–0.97)	0.032 *	0.0	Figure 5	No
Time (minutes) to first requirement of anesthetic intervention	Chua et al. [18], Fettes et al. [19], Leo et al. [20], Lim et al. [21], Lim et al. [22], Wang et al. [26] and Ojo et al. [27]	465	−49.31 (−53.96 to −44.66)	<0.001 *	67.6	Figure 6	No
Delivered dosage (milligrams per hour) of local anesthetic (bupivacaine equivalents)	Capogna et al. [17], Fettes et al. [19], Leo et al. [20], Lim et al. [21], Lim et al. [22], Salim et al. [23], Sia et al. [24], Wong et al. [25], Wang et al. [26] and Ojo et al. [27]	896	−1.71 (−1.88 to −1.55	<0.001 *	78.1	Figure 7	No
Maternal satisfaction (visual analog scale from 0 to 100)	Leo et al. [20] Lim et al. [21] Lim et al. [22] Sia et al. [24] Wong et al. [25]	340	−6.95 (−7.77 to −6.13)	<0.001 *	0.0	Figure 8	No
Duration (minutes) of first stage of labor analgesia	Fettes et al. [19], Leo et al. [20], Lim et al. [22], Salim et al. [23], Sia et al. [24], and Wong et al. [25]	445	−7.47 (−33.41 to 18.48)	0.573	0.0	Figure 9	1.94 (0.892) for PCEA ([20], [23], [24] and [25]) −61.31 (0.074) for non-PCEA ([19] and [22])

Abbreviations: CI, confidence interval; OR, odds ratio; PCEA, patient-controlled epidural analgesia. * *p*-Value <0.05.

## Supplementary Materials

The following are available online at https://www.mdpi.com/1660-4601/17/19/7082/s1, Table S1: Summary of risk of bias assessment and relevant characteristics of eligible studies, Table S2: Quality assessment results of eligible studies.
