# Supplementary material for: A Meta-Analysis of Comparing Intermittent Epidural Boluses and Continuous Epidural Infusion for Labor Analgesia
Source: Int J Environ Res Public Health. 2020 Sep 27;17(19):7082. doi: 10.3390/ijerph17197082 (PMC7579642; doi:10.3390/ijerph17197082)
Supplement: Supplementary file 1 [file ijerph-17-07082-s001.pdf]

Supplementary Table S1. Summary of Risk of Bias Assessment and Relevant Characteristics of Eligible Studies.

Study	Total Number of Parturients	Parity	Medication for Epidural	The Epidural Maintenance Regimen Used CEI	The Epidural Maintenance Regimen Used IEB	Labor Process, n (%) for Labor Analgesia Mode	Quality Assessment
Capogna et al. [17]	145	Nulliparous	Levobupivacaine, 0.0625%–0.125%; sufentanil, 0.5μg/mL	10 mL/h (0.0625%) *PLUS* PCEA (5 mL bolus, 10minute lockout, 0.125%) (n = 70)	10 mL (0.0625%) bolus every hour *PLUS* PCEA (5 mL bolus, 10 minute lockout, 0.125%) (n = 75)	Spontaneous labor; oxytocin use not reported	Low risk
Chua et al. [18]	42	Nulliparous	Ropivacaine, 0.1%; fentanyl, 2 μg/mL	5 mL/h (n = 21)	5 mL bolus every hour (n = 21)	Spontaneous labor; preanalgesia oxytocin; CEI, 8 (38); IEB, 9 (43)	Low risk
Fettes et al. [19]	40	Nulliparous	Ropivacaine, 0.2%; fentanyl, 2 μg/mL	10 mL/h (n = 20)	10 mL bolus every hour (n = 20)	Labor induction; CEI, 12 (60); IEB, 14 (70)	Low risk
Leo et al. [20]	62	Nulliparous	Ropivacaine 0.1%; fentanyl 2 μcg/mL	5 mL/h *PLUS* PCEA (5 mL bolus, 10 minute lockout) (n = 31)	5 mL bolus every hour (or 30 minutes after successful PCEA dose) *PLUS* PCEA (5 mL bolus, 10 minute lockout) (n = 31)	Preanalgesia oxytocin; CEI, 10 (32); IEB, 15 (48)	Low risk
Lim et al. [21]	60	Nulliparous	Levobupivacaine, 0.1%; fentanyl, 2 μg/mL	10 mL/h (n = 30)	5 mL bolus every 30 minutes (n = 30)	Preanalgesia oxytocin; CEI, 4 (13); IEB, 9 (30)	Low risk
Lim et al. [22]	50	Nulliparous	Ropivacaine, 0.1%; fentanyl, 2 μg/mL	10 mL/h (n = 25)	2.5 mL bolus every 15 minutes (initiated 7.5 minutes after dosage of subarachnoid) (n = 25)	Spontaneous labor; preanalgesia oxytocin; CEI, 10 (40); IEB, 5 (20)	Low risk
Salim et al. [23]	127	Nulliparous	Bupivacaine, 0.125%–0.25%; fentanyl, 2 μg/mL	8 mL/h (0.125%) *PLUS* PCEA (3 mL bolus, 20 minute lockout) (n = 63)	10 mL bolus every hour (0.25%) (n = 64)	Labor induction; CEI, 17 (27); IEB, 14 (22)	Low risk
Sia et al. [24]	42	Nulliparous	Ropivacaine, 0.1%; fentanyl, 2 μg/mL	5 mL/h *PLUS* PCEA (5 mL bolus, 10 minute lockout) (n = 21)	5mL bolus every hour (or 1 hour after successful dosage of PCEA) (n = 21)	Spontaneous labor; preanalgesia oxytocin; CEI, 5 (24); IEB, 7 (33)	Low risk
Wong et al. [25]	126	Parous	Bupivacaine, 0.625%; fentanyl 2, μg/mL	12 mL/h *PLUS* PCEA (5 mL bolus, 10 minute lockout) (n = 63)	6 mL bolus every 30 minutes *PLUS* PCEA (5 mL bolus, 10 minute lockout) (n = 63)	Labor induction; CEI, 63 (100); IEB; 63 (100)	Low risk

Wang et al. [26]	186	Nulliparous	Bupivacaine,0.08%; fentanyl, 0.4μg/ml	10ml/60min *PLUS* PCEA (5 ml bolus, 30 minute lockout) (n = 62)	5ml/30min *PLUS* PCEA (5 ml bolus, 30 minute lockout) (n = 62)	Preanalgesia oxytocin; Labor induction; CEI, 18 (29); IEB; 12 (19)	Low risk
Ojo et al. [27]	120	Nulliparous /Parous	0.1% ropivacaine with 2 μg/mL fentanyl	8 mL/h *PLUS* PCEA (8 mL boluses with a 10-minute lockout) (n = 59)	6-mL programmed intermittent epidural boluses every 45 minutes *PLUS* PCEA(8 mL boluses with a 10-minute lockout) (n = 61)	Preanalgesia oxytocin; Labor induction; CEI, 44 (75); IEB; 46 (75)	Low risk

Supplementary Table S2. Quality Assessment Results of Eligible Studies.

Questions	Capogna et al. [17]	Chua et al. [18]	Fettes et al. [19]	Leo et al. [20]	Lim et al. [21]	Lim et al. [22]	Salim et al. [23]	Sia et al. [24]	Wong et al. [25]	Wang et al. [26]	Ojo et al. [27]
Was the method of randomization adequate?	Positive	Negative	Positive	Negative	Negative	Positive	Positive	Positive	Positive	Positive	Positive
Was the treatment allocation concealed?	Positive	Positive	Positive	Positive	Negative	Positive	Positive	Positive	Positive	Positive	Positive
Was the patient blinded to the intervention?	Positive	Positive	Positive	Positive	Negative	Positive	Inconclusive	Positive	Positive	Inconclusive	Positive
Was the care provider blinded to the intervention?	Positive	Positive	Positive	Positive	Inconclusive	Positive	Inconclusive	Positive	Positive	Inconclusive	Positive
Was the outcome assessor blinded to the intervention?	Positive	Positive	Positive	Positive	Positive	Positive	Inconclusive	Positive	Positive	Inconclusive	Positive
Was the dropout rate described and acceptable?	Positive	Positive	Positive	Positive	Positive	Positive	Positive	Positive	Positive	Negative	Positive
Were all randomized participants analyzed in their allocated group?	Positive	Positive	Positive	Positive	Positive	Positive	Positive	Positive	Positive	Positive	Positive
Are reports of the study free of suggestion of selective outcome reporting?	Positive	Positive	Positive	Positive	Positive	Positive	Negative	Positive	Positive	Positive	Positive
Were the groups similar at baseline?	Positive	Positive	Positive	Positive	Positive	Positive	Positive	Positive	Positive	Positive	Positive
Were cointerventions avoided or similar?	Positive	Positive	Positive	Positive	Positive	Positive	Positive	Positive	Positive	Positive	Positive
Was the compliance acceptable in all groups?	Positive	Positive	Negative	Positive	Positive	Positive	Positive	Positive	Positive	Positive	Positive
Was the timing of the outcome assessment similar?	Positive	Positive	Positive	Positive	Positive	Positive	Positive	Positive	Positive	Positive	Positive
